# Valproate Annual Risk Acknowledgement Form: Evaluating Compliance and Creating Digital Solutions

**DOI:** 10.1192/bjo.2025.10352

**Published:** 2025-06-20

**Authors:** Neelam Choudhary, Amitav Narula, Renju Joseph, Brian OConnor, Perry Otchere

**Affiliations:** Black Country Healthcare NHS Foundation Trust, Dudley, United Kingdom

## Abstract

**Aims:** Valproate is a commonly prescribed drug in neurology and psychiatry, licensed for epilepsy as an anticonvulsant and in bipolar disorder as a mood stabiliser. Valproate is highly teratogenic with evidence suggesting that use in pregnancy leads to neurodevelopmental disorders (approximately 30–40% risk) and congenital malformations (approximately 10% risk). Consequently valproate must not be used in females of childbearing age unless conditions of pregnancy prevention programme (PPP) are met.

To aid the monitoring of risk, the Annual Risk Acknowledgment (ARA) form for valproate forms a key part of the UK’s valproate pregnancy prevention programme. It documents the patient’s awareness of risk, reinforces the PPP, promotes informed decision making and vitally ensures compliance monitoring to ensure prescribers are following national guidelines.

To measure compliance with completion of the ARA form for all adult female patients (18–65) prescribed valproate within our local mental health outpatient and inpatient services.

To devise digital solutions for record keeping and reminders with the aim to support clinicians to complete the forms in a thorough and timely manner.

**Methods:** With support from pharmacy we retrieved a list of female patients prescribed valproate in our locality, which served as a central valproate register. We examined patient records to determine whether an ARA form was on their records and if the form was completed and up to date. We then produced a list of patients who required renewal/completion of the form.

The team met with Information technology system provider (RIO) to discuss creation of a digital central valproate register and using digital clinical reminders on patient’s records to notify clinicians when the form was due for renewal.

**Results:** Reminders were sent to relevant clinicians/teams, requesting them to complete the required ARA form at earliest opportunity. The data from the central valproate register was shared with the RIO team who agreed to transfer this data to the electronic records intervention list in order to create digital version. They then agreed to create a valproate tab in patient’s records, and link the ARA form to the tab. This link up will automatically act as trigger to warn clinicians that the ARA form is due for completion.

**Conclusion:** This project has created a central database for local service users who are on valproate. By doing so it has facilitated the tracking of ARA forms for the clinicians. Creation of automatic reminders will further help clinicians in completing the required form in timely manner.

